# Coating and Functionalization Strategies for Nanogels and Nanoparticles for Selective Drug Delivery

**DOI:** 10.3390/gels6010006

**Published:** 2020-02-04

**Authors:** Filippo Pinelli, Giuseppe Perale, Filippo Rossi

**Affiliations:** 1 Dipartimento di Chimica, Materiali e Ingegneria Chimica “Giulio Natta”, Politecnico di Milano, via Mancinelli 7, 20131 Milan, Italy; filippo.pinelli@polimi.it; 2 Faculty of Biomedical Sciences, University of Southern Switzerland (USI), Via Buffi 13, 6900 Lugano, Switzerland; giuseppe.perale@usi.ch; 3 Ludwig Boltzmann Institute for Experimental and Clinical Traumatology, Donaueschingenstrasse 13, 1200 Vienna, Austria

**Keywords:** nanoparticle, nanogel, polymer functionalization, selectivity

## Abstract

Drug delivery is a fascinating research field with several development opportunities. Great attention is now focused on colloidal systems, nanoparticles, and nanogels and on the possibility of modifying them in order to obtain precise targeted drug delivery systems. The aim of this review is to give an overview of the main available surface functionalization and coating strategies that can be adopted in order to modify the selectivity of the nanoparticles in the delivery process and obtain a final system with great targeted drug delivery ability. We also highlight the most important fields of application of these kinds of delivery systems and we propose a comparison between the advantages and disadvantages of the described functionalization strategies.

## 1. Introduction

Every time a drug has to be administered the method and the timing of the process can be very impactful on its efficacy [1,2]. Indeed, every drug has its own concentration range and targeted body area in which the maximum benefits can be obtained. At present, an exciting challenge for the scientific community is represented by the ability to reach the optimum conditions by exploiting new formulations, products, and materials that can transport a pharmaceutical compound in the body and achieve the desired therapeutic effect [3]. All of these issues are commonly identified in the drug delivery field, the focuses of which are mainly the quantity of pharmaceutical product administered, as well as the manner and duration of the delivery.

There is a wide range of technologies that can be applied to achieve this aim, and they can influence different aspects of the process: drug release rates, drug adsorption in the body, the timing of the adsorption, and so on [4]. At the same time, each of these aspects can be realized in different ways: herein our attention is focused on the drug’s delivery and its release, since these are the main steps that can be influenced by the framework in which the drug is loaded. 

The drug release can take place because of diffusion gradient, degradation of the support in which the drug is trapped, swelling behavior, or mechanisms based on affinity [4]. In recent years, great consideration has been given to nanoparticles as a drug delivery platform, particularly polymeric nanoparticles [5], which are colloidal particles with dimensions between 1 and 100 nm that are very interesting because of their properties such as biocompatibility, biodegradability, water solubility, and non-toxicity [6]. Many preparation strategies are available [7], such as: solvent evaporation [8,9], emulsification [10], or solvent diffusion [11]. The synthesis and formulation methodology strongly affect the properties of the final product [12] such as the size, the mesh of the particle, its swelling behavior, the degradability of the product, and its solubility, which are all very important for drug delivery.

Great attention has been given to these colloidal formulations because of the great potential that the polymeric nanoparticles have as drug carriers [5,13]. Nanoparticle design requires great attention on two key aspects: the first is the incorporation and preservation of the drug inside the colloidal system to guarantee the specific desired action inside the human body. Generally, nanoparticles are able to guarantee the drug’s protection and stabilization. Moreover, they are able to take in a high drug loading [14,15]. There are several ways through which the drug can be loaded with the nanoparticle: it can be entrapped inside it (e.g., encapsulation), it can be coated on the surface of the particle, or it can be chemically linked with the particle itself. The second key point in nanoparticle design is the necessity to provide the nanoparticles with specific properties so that their interaction with the external environment in the human body increases the targeting action toward specific sites.

This review focuses on the available possibilities to improve this second design aspect through the surface modification (coating) of pre-existing nanoparticles. Clearly there is no one best way, because the synthesis method is strongly related to the application for which the particle is intended. In fact, this kind of drug delivery system can be applied for treating a wide variety of diseases including tumor diseases [16,17], but also inflammation diseases (e.g., in the brain area) or diseases coming from injuries (e.g., spinal cord injuries) [18].

Currently the heart of drug delivery research is focused on so-called targeted drug delivery—a branch of drug delivery for which the focus is on being able to reach one single specific area of the body, increasing drug efficacy and limiting side effects [3].

## 2. Nanoparticles and Nanogels

### 2.1. Definition and Properties

As mentioned previously, in the field of drug delivery great attention is currently focused on nanoparticles, which are commonly defined as objects ranging in size around 1–100 nm with possible different natures [19]. All nanoparticles are characterized by a very high surface-area-to-volume ratio [20], which ensures their specific properties and is the reason for the great importance played by the surface of a nanoparticle. In fact, the surface properties and the surface functionalization, which can be realized with metal ions, small molecules, surfactants, or polymers, are key elements that strongly influence the final properties of the system.

In addition to the surface, two other key elements of a nanoparticle that have to be considered are the shell and the core. The shell is a layer of material that is chemically different from the core, which is instead the inner portion [20]. As reported by Christian and co-workers [20], an example of a shell structure can be found by considering the case of iron nanoparticles, which rapidly form layers of iron oxide at their surface after preparation: in many cases, the iron oxide does not penetrate the whole particle and therefore the final system presents an outer layer (iron oxide) that is clearly different from the inner core (pure iron) [21]. The core is the center of the nanoparticle, and it is its inner portion. Commonly, even though the surface and the layer can have a strong influence on the final characteristics of the system, the properties of interest to the scientific community are dominated by the specific properties of the core and its composition. The great importance that is given to the nanoparticles is due to their characteristics, such as biocompatibility, biodegradability, and non-toxicity [2,22]. 

Among the available typologies of nanoparticles and colloidal systems, nanogels are upcoming structures with specific and interesting properties [23]: they are commonly defined as nanoparticles comprised of a three-dimensional network, either chemically or physically cross-linked, that in most cases has a polymeric nature. Many times, they are even simply defined as particles of gel of any shape with an equivalent diameter approximately 1 to 100 nm [24]. Their formulation is very close to biological tissues because of the high water content and carbon-based composition: this guarantees a high degree of biocompatibility and biodegradability [25]. The most important properties of nanogels are firstly the large surface area and the good structure stability. Moreover, they have a high drug-loading capacity and an ability to swell that makes them very suitable for drug delivery applications [26].

### 2.2. Synthesis and Formation Methods

Nanoparticles preparation can be realized through different methods. Generally, two main approaches are available: the “top down” method and the “bottom up” method [20]. The first methodology obtains nanoparticles by “cutting” larger pieces of a material until a nanoparticle remains. In contrast, the “bottom up” method allows nanoparticles to be produced by growth from simple molecules: the growth can be controlled by modifying the concentration, the operating conditions, or by functionalizing the particle itself. Considering the specific case of polymeric nanoparticles [27], they are usually prepared by emulsion polymerization. The first step of this methodology is usually the preparation of an emulsion of monomer using a non-solvent and a surfactant and then a free-radical polymerization is initiated by a water-soluble initiator. Another common method is co-precipitation [28], where the simultaneous occurrence of nucleation, growth, coarsening, and agglomeration processes takes place. This method is quite simple and rapid, and it allows a good control of particle size and composition. However, this methodology has batch-to batch reproducibility problems and it does not work well if the reactants have very different precipitation rates. The methods explained above are not the only available methods in the literature: other examples are sputtering [28], which is the ejection of atoms from the surface of a material by bombardment; the sol–gel method [29], where the sol (solution) evolves toward the formation of a gel until the formation of a nanoparticle; and others [27].

As in the case of nanoparticles, various strategies are also available for the preparation of cross-linked nanogels [30]. First of all, we have to consider the synthesis through chemical reaction that involves a heterogeneous polymerization of low-molecular-weight monomers or the cross-linking of polymer precursors [31,32]. The first method uses radical polymerization promoted with specific initiators that guarantee the possibility of controlling and tuning the process. The latter method instead obtains nanogels by linking polymer precursors, and they are obtained by the self-assembly of amphiphilic or triblock copolymers. The nanogel formation can also be promoted using physical cross-linking: the synthesis is usually realized under mild conditions and can exploit different interactions as electrostatic, host–guest, and hydrophobic/hydrophilic [33,34]. In addition to these two classical families of synthesis methods, nanogels can be obtained with innovative technologies such as PRINT (particle replication in non-wetting templates) technology [25] or molecular imprinting [35]. The PRINT technology allows monodisperse particles to be obtained from a wide range of matrix materials, and with this method it is possible to precisely control the particle size, composition, shape, and many other parameters. Molecular imprinting instead makes it possible to create template-shaped cavities in polymer matrices, and thanks to this the final nanoparticle is suitable for different purposes. 

Obviously, these synthesis procedures are not the only ones available: many other methods to obtain final nanogel nanoparticles can be found in the literature [36].

Despite the synthesis method adopted, the key point in nanoparticle and nanogel formulation is to be able to impose specific properties upon the systems, to tune their behavior, and to promote the nanoparticle formation. 

### 2.3. Polymer Functionalization for Nanoparticles Synthesis

In order to obtain final nanogels nanoparticles the cross-linking formation among various polymers is pivotal: the presence of characteristic chemical groups or specific molecules can have important role in the synthesis method [37]. Obviously, the choice of polymers is not trivial [38]: on one hand natural polymers can be used in order to have a final product with marked biocompatibility. On the other hand synthetic polymers can be used to set mechanical properties, degradation, or to tune the system behavior. A very important working technique is currently represented by the possibility of grafting different molecules or functional groups over existing polymers: in this review we give a brief overview of the available polymer functionalization methods as illustrated by Blasco and co-workers [39]. 

The authors have identified the following classes of reactions:

- The activation of esters under mild conditions to form amide bonds. This technique represents one of the most interesting strategies for polymer functionalization: esters [40] are very flexible for different kinds of functionalization while amides are very versatile linkages in organic chemistry thanks to their stability in chemical environments and compatibility with different functionalities.
- Click chemistry, which allows the introduction of compatible click functional groups in pre-existing molecules that are able to activate molecules and polymers [41]. This process is usually performed with copper(I)-catalyzed azide–alkyne cycloaddition (CuAAC process), but it can also be realized with a copper-free strain promoted azide–alkyne process to overcome the cytotoxicity of the CuAAC reaction [42].
  This technique is stereospecific and generates non-toxic byproducts that can be easily removed. Moreover, functionalization with azide and alkyne groups offers the possibility to link very different molecules and improve material properties and compatibility.
- Thiol chemistry represents another important process in this field thanks to the great available functional thiols and reactions in which they can participate [43,44]. They have radical- and light-mediated reactivity with carbon–carbon double bonds, and this allows the advantages of click reactions to be combined with those of photoinitiated reactions [45]. In this way, it is possible to obtain quantitative yields, high reaction rates, and easy product recovery. At the same time, the method based on electrophile and nucleophile interactions allows operation in mild reaction conditions, has great compatibility with different functional groups, high conversion, and very good possibilities of application in different fields such as biomedicine.
- The addition of alcohols, amines and thiols to isocyanates [46] represents a possible effective strategy in polymer functionalization thanks to fast reaction kinetics, the stability of isocyanates toward radicals, and the good yields. However, their application is limited by the toxicity of isocyanate and the instability of final mixtures containing isocyanate and polymer mixtures.
- Imine [47] and oxime [48,49] linkages have an important role in the field of macromolecular modification reactions. They allow the bond reversibility in the case of imine linkage thanks to the equilibrium of this molecule and in the case of oxime under aqueous acid conditions. This functionalization method can be tuned according to the desired application: the obtained linkage can be hydrolytically stable or unstable according to the need, in order to preserve the functionalization or to release the grafted molecule.
- Ring-opening reactions are a classical and versatile method in polymer science [50]. They allow the ring of strained heterocycles to be opened and for desired heteroatoms to be introduced on the polymer backbone [51,52]. The most commonly employed functional groups in this kind of synthesis are the epoxides, but in recent years ring-opening modifications involving aziridines and azlactones have been reported.
- Multicomponent reactions (MCRs) [53] are an upcoming synthesis methodology which are of great interest because of their atom economy. Their advantage lie in their ability to introduce a high degree of functional complexity in a single modification step [54]. These kinds of reactions include isocyanide-based MCRs, non-isocyanide-based MCRs, and MCRs catalyzed by organometallic species.

In order to realize polymer functionalization, together with the illustrated methodologies, there are non-covalent interactions available: these include van der Waals, hydrogen bonding, and charge transfer interactions that allows physical modifications to be introduced on the nanoscale of the structure. However, all the methodologies explained above are effective methods to obtain final reactive polymers that can be applied in the synthesis of drug delivery systems. In the following section we explain the importance of these kinds of devices and their applications.

## 3. Targeted Drug Delivery: Selectivity of the Delivery Process and Its Applications

The possibility of delivering a drug to a specific target site inside the human body presents many advantages [55]. First of all, in this way it is possible to localize the drug in the body area of interest and to extend the release period. Moreover, the interaction of the drug with other tissues and the risk of side effects can be limited: because of this, a more uniform drug distribution is provided and a smaller quantity of drug is needed because of the limited losses of pharmaceutical product elsewhere in the body [56,57]. In order to achieve the targeted delivery, more difficult synthesis methods are required, which commonly involve more steps for the synthesis of the final nanoparticle, also including much more time and higher costs.

In the literature, the targeted drug delivery strategies are commonly divided into two types: passive targeting and active targeting [16,17]. Passive targeting exploits the so-called enhanced permeability and retention effect, and so its efficacy it is based on the accumulation of the nanoparticles in specific tissues: it is clear how the success of this technique is based on the circulation time. Instead, active targeting improves the effects of passive targeting, making the delivery system more specific to a target tissue. Obviously, if properly designed, this last targeting method can be much more effective and convenient. Different methods that are able to increase drug delivery selectivity are reported in the literature: liposomes are the most common strategy used for targeted dug delivery [58,59]. Liposomes are highly bio-compatible and bio-degradable, and are non-toxic, non-hemolytic, and non-immunogenic [59]. They are often PEGylated to elongate the half-life of the structure while maintaining the targeting effect.

Other commonly used systems are represented by micelles and dendrimers [60]: they have polymer-based structures and they show great ability to target specific sites. In this review, the main focus will be on a different upcoming possibility to achieve the goal of targeted drug delivery: starting from existing nanoparticles or formulations, the idea is to functionalize their surface (coating the nanoparticle) with other molecules or substances that are able to influence the drug carrier’s behavior once the coated nanoparticle is inside the human body, ensuring a certain selectivity for a specific target [61,62,63]. The choice of the molecule or the system to be used as coating is not trivial: many elements have to be considered [64,65]. First of all, the target tissue: it is essential to determine which classes of compounds can be useful in order to target a specific area. In order to do that, the knowledge in the biological field is essential because it is necessary to be aware of the biological mechanisms that take place in this specific area of the human body. For example, there are tissues that cannot be easily accessed because of the presence of biological barriers [65] inside human body (e.g., the central nervous system, CNS) [66]: being conscious of the chemical characteristics of human body compounds that are able to overcome these barriers can be the key to finding a coating able to guarantee the proper selectivity for the drug delivery process [67]. 

There are other important aspects to consider: the coating used has to be biocompatible, and there must be an available method to place it on the nanoparticle. Furthermore, the quantity of the coating used is not random: in fact, the presence of these molecules on the surface affects the dimensions of the system, and a higher amount of coating can cause a reduced internalization inside the cells because of the oversized steric hindrance [68,69]. 

Moreover, the final product should be stable and not break down during the delivery. These kinds of targeted drug delivery systems can have different applications. The most important field in which they can be applied is the treatment of cancerous tumors [70]. In this specific case, the passive method of targeting is promoted by the enhanced permeability and retention (EPR) effect [71]. This is a situation typical of tumors by which molecules of certain sizes (typically liposomes or nanoparticles) have the tendency to accumulate in diseased tissues much more than in normal tissues. Because of this, a high percentage of the targeted drug delivery systems used are able to successfully reach the sites of interest for successful treatment to take place. 

Other important applications are the treatment of diabetes [71] and cardiovascular diseases [72]: in the case of diabetes, new formulations are being explored in order to find less-invasive routes for insulin delivery and improving the drug delivery [73]. 

Instead, in the case of cardiovascular diseases [72,74] the use of a targeted drug delivery system plays an important role in the development of regenerative medicine and in the shift from conventional approaches to new therapies that are able to manage these kinds of illnesses [74].

Another important field of application is the targeting of the central nervous system in order to treat the diseases of this neuronal tissue, such as spinal cord injuries (SCIs), meningitis, encephalitis, or degenerative diseases such Alzheimer’s, Parkinson’s, and others [75]. The big challenge in treating such CNS diseases has long been the difficulties in surpassing the natural CNS protective barriers: the blood–brain barrier (BBB) and the blood–cerebro-spinal fluid barrier (BCSB). Nanotechnologies and nanoparticles [75] provide the possibility of delivery systems able to deliver drugs across these barriers, improving therapeutic effect and reducing the risks compared with conventional treatments.

In the following table (Table 1) we have highlighted the main applications of nanoparticles as drug delivery systems and their advantages.

**Figure 1:**
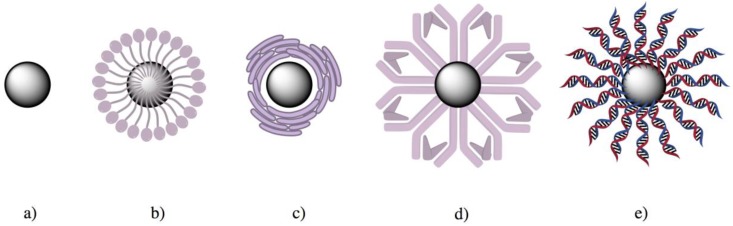
Examples of surface modification over an existing nanoparticle (**a**). The schematization in the figure represents the functionalization using (**b**) polymers, (**c**) cell membrane, (**d**) proteins, or (**e**) hybridized DNA structure.

In Figure 1 we have schematized some coating possibilities over a preformed nanoparticle:

In the following sections we analyze different compounds that can be used as coating and the techniques through which they can be placed on the colloidal particle.

## 4. Available Strategies to Coat Nanoparticles

### 4.1. Polymers

Polymers, commonly large linear or branched molecules with long chains, can include different functional groups [76]. Due to their peculiar structures, they offer great possibilities for the coating of nanoparticles and drug delivery/selectivity enhancement. 

When located on a particle’s surface they are able to give them specific properties and functionalities: the surface modification is in fact crucial for the biological response and to improve the therapeutic efficiency [77,78,79]. Examples of polymers that can be used include polyethylene glycol, polycaprolactone, polysorbate, polylactic acid, dextran, chitosan, and many others [78,79,80,81]. A great advantage of using polymers is the possibility of modifying their chain’s structure, and tune their characteristics by doing so. In this contest, Mauri and co-workers have proposed to use specific modified polymers in order to coat existing nanogels (NGs) [82]: mPEG linked with imidazole (NG-Im) and mPEG modified with terminal carboxyl group (NG-COOH). The aim of these coatings was to be able to target the tissues of the CNS and have a good selectivity in order to fight spinal cord injuries. These coating molecules have been grafted onto pre-existing nanoparticles: the functionalization of the nanogel takes place because a covalent link is formed between the polytethylenimine (PEI) molecules present in the cross-linked framework of the nanogel and the polymer chains used as coating. The great advantage of this coating method is firstly the physical-chemical properties of the molecules [83] used to coat the nanoparticle: they are highly soluble, non-toxic, biocompatible, and non-immunogenic. Moreover, this kind of functionalization plays an important role in preventing surface agglomeration [67]. 

**Figure 2:**
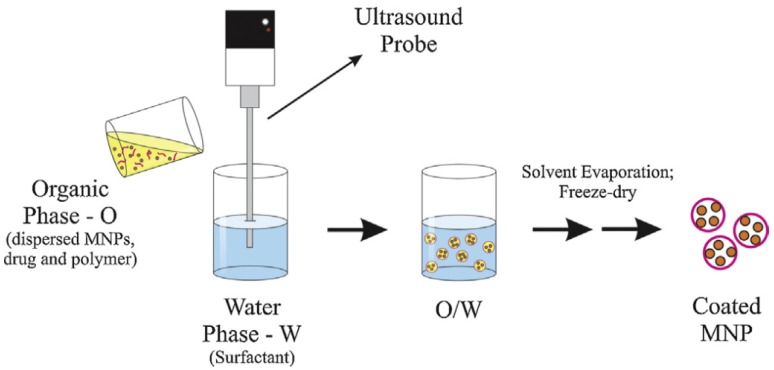
Set-up used for a simple emulsion evaporation method to obtain magnetic nanoparticles (MNPs). In this case, an oil-in-water (O/W) emulsion is exemplified. Reprinted with permission from Elsevier [84].

Tests in purified microglia cultures show how the coatings alter the NGs’ uptake inside the cells: in that specific case the NG-COOH system was less internalized by microglia with respect to a non-coated NG because of steric hindrance. The NG-Im system instead showed higher permeability inside the cell membrane. In fact, as mentioned previously, the size of the nanoparticle strongly influences the possibility of interaction with the cell: dimensions of up to 100–200 nm allow the internalization through receptor-mediated endocytosis, while phagocytosis is the process of uptake for larger particles [68,82]. Tenório et al. proposed similar coating methodologies for magnetic nanoparticles [84]. Magnetic nanoparticles are an up-and-coming delivery system that are usually composed of an iron oxide core with an external coating of organic materials such as fatty acids, polysaccharides, or polymers [85]. In this specific case, the presence of a polymer surface coating is essential for a successful target delivery. In fact, the magnetic nanoparticles can be led to and maintained in the target tissue through an external magnetic field [86]: the presence of the polymer coating has an essential role in protecting drugs until reaching the cancer cells and preventing nanoparticles’ irreversible aggregation, guaranteeing their stabilization. Moreover, they protect the particles’ core from oxidation [85]. There are different available techniques [87,88] in order to realize this type of coating. The encapsulation can be realized through co-precipitation. Another possibility is the encapsulation via simple emulsion evaporation, which first requires the formation of a simple emulsion, then the solvent evaporation permits the precipitation of the polymers, and then the formation of the particles. Encapsulation via double emulsion evaporation is a similar process obtained by two emulsification processes. Unlike the previously described processes, another possibility is layer-by-layer encapsulation, which is a process based on the electrostatic attraction between oppositely charged elements: this is basically an adsorption of the polymers on the particle surface. Figure 2 below presents a schema of the emulsion evaporation method for the synthesis of magnetic nanoparticles.

### 4.2. An Organic Solution: Cell Membrane Coating

**Figure 3:**
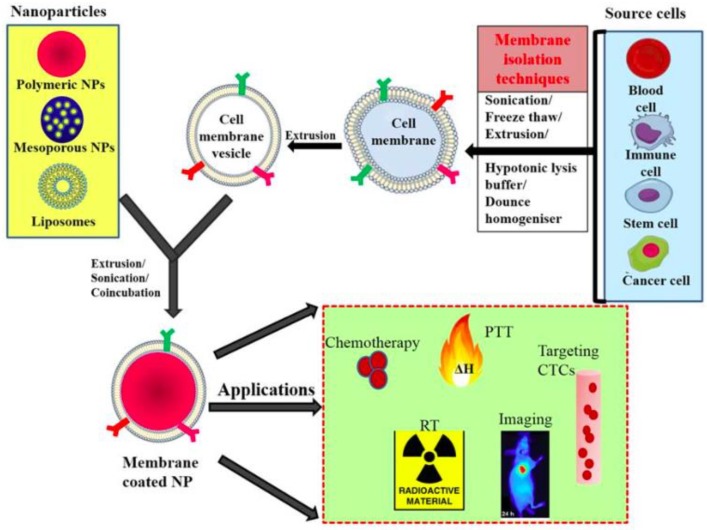
General scheme of preparation of membrane-coated nanoparticle and its biomedical applications; membranes isolated from different source cells by various methods and coating this onto core NPs by coincubation, sonication, or extrusion. CTC: circulating tumor cell; NP: nanoparticle; PTT: photothermal therapy; RT: radiotherapy. Reprinted with the permission from MDPI [96].

Another coating methodology that has recently gained increasing interest uses natural cell membranes [89,90,91]: these are collected intact from natural cells and spread on pre-existing nanoparticles. It is clear how this methodology guarantees that the resulting system has highly tunable characteristics and properties typical of the cell membrane used. Moreover, the coated NP displays a bi-layered medium that it is useful for trans-membrane protein anchorage. There are two available techniques to realize this surface coating: the first is the so called “top-down” approach [92], and the second is microfluidic electroporation [93]. In the former, the intracellular contents are removed from the cells through a hypotonic treatment and then cell-membrane vesicles are obtained by extruding the emptied washed cells with a porous polycarbonate membrane. Finally, cell-membrane-derived vesicles are fused with the nanoparticles through extrusion. In the latter case, the nanoparticles and the cell-membrane-derived vesicles are injected into the system and electric pulses between two electrodes can promote the entry of the nanoparticles inside the cell membranes. Different types of cell membranes can be used: red blood cells (RBCs) are an example of a natural long-circulation delivery system [94,95] and therefore great attention has been given to them. Other possibilities are cancer cell membranes, stem cell membranes, fibroblast cell membranes, platelet membranes, and many more. The advantages of this coating methodology are closely related to the type of membrane used: RBC membranes ensure a long circulation time but, at the same time, have a limited ability to target tumors. In other cases (cancer cell membrane, platelet membrane, and similar) the resulting systems have usually shorter blood circulation time, but ensure very good targeting delivery, according to the type of membrane used [91]. Moreover, a central aspect is that these nanoparticles have reduced compatibility issues because the membranes used as coating are recognized by the immune system and so are not immunogenic [89]. In Figure 3 we report a scheme representing the process of membrane-coating a nanoparticle with the possible practical applications.

### 4.3. Proteins and Antibodies: A Very Promising Possibility

**Figure 4:**
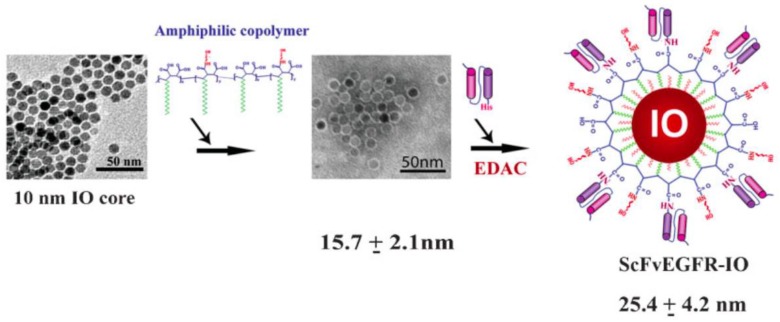
Construction of ScFvEGFR (single-chain variable fragment of the epidermal growth factor receptor)-IO (iron-oxide) nanoparticles. Uniform 10 nm IO nanoparticles were coated with amphiphilic copolymers modified with short PEG chains. ScFvEGFR proteins were conjugated to the IO nanoparticles mediated by ethyl-3-dimethyl amino propyl carbodiimide (EDAC). Reprinted with the permission from Wiley [97].

Proteins are another class of molecules that can be used in order to realize nanoparticle coatings [17]. There are several types of these compounds in nature, and some of them are very interesting in targeted drug delivery. Particularly, great attention is given to the antibodies, as they can be used to functionalize the nanoparticle surface, and they are able to guarantee high levels of selectivity. Yang and co-workers reported an example of this possibility [97]. They propose to use a single-chain anti-EGFR (epidermal growth factor receptor) [98] antibody to functionalize quantum dots or magnetic iron oxide nanoparticles. The resulting system is a compact nanoparticle that is reported to be internalized by EGFR-expressing cancer cells. The methodology used to coat the nanoparticles in this specific case is quite simple: first, nickel nitrilotriacetic acid (Ni-NTA) is conjugated with the quantum dots through a covalent linkage between the amine group of Ni-NTA and the carboxyl group of the amphiphilic polymer on the surface of the quantum dots. Then, the His-tagged (polyhistidine tag) ScFvEGFR (single-chain variable fragment of the epidermal growth factor receptor) proteins are added to the system and the interaction between the His-tag and the nickel molecule of NTA determines the formation of a stable drug delivery system. Generally the linkage method is performed using His-tagged proteins that are able to link with properly designed nanoparticles [99]. This working method shows great opportunities for in vivo tumor imaging and the delivery of therapeutic agents thanks to the usage of the antibodies. Given its importance inside the family of human proteins, it is important to determine the possibility of using albumin as a coating material [100]. Generally, this type of coating is realized with a methodology similar to the one previously explained. The great advantage of this molecule is the possibility of overcoming the opsonization problems typical of drug delivery systems due to its physiological peculiarity in blood circulation [17,100]. Figure 4 presents an example of the synthesis of a specific type of protein-coated nanoparticle.

### 4.4. Novel Strategy: Hybridized DNA Structure

Hybridized DNA structures are very promising molecules that can be used as coating agents [101,102]. In fact, they can guarantee precise targeted delivery, especially on cancer cells, with high affinity and specificity [103]. In the literature, many examples of aptamers [104] (short single-stranded DNA or RNA oligonucleotides) used as nanoparticle coatings have been reported. Their advantages are first that they are very specific, non-immunogenic, and biocompatible. Sun and co-workers reported the conjugation of a hybridized DNA structure with superparamagnetic iron oxide nanoparticles (SPIONs) in order to obtain targeted drug delivery systems [105]. This hybridized DNA structure is composed of an aptamer, an intercalated motif (i-motif), and ds-DNA: the i-motif DNA is a type of deoxyribonucleic acid with a four-stranded quadruplex structure, whereas ds-DNA is the double stranded DNA. The aptamer guarantees active and magnetic target-specific delivery, usually to cancer cells. The i-motif DNA instead plays a key role in the fast and efficient release of the drug at the target site. The synthesis of this type of coating is usually obtained by adding the proper compounds to a system under stirring (in the analyzed case, N-(3-dimethylaminopropyl)-N′-ethylcarbodiimide (EDC) and N-hydroxysuccinimide (NHS) and then N-terminated single-stranded Apt-gc34). Then, the unreacted DNA is removed by centrifugal filtration and the formation of DNA oligonucleotides conjugated SPIONs is verified with gel electrophoresis. 

**Figure 5:**
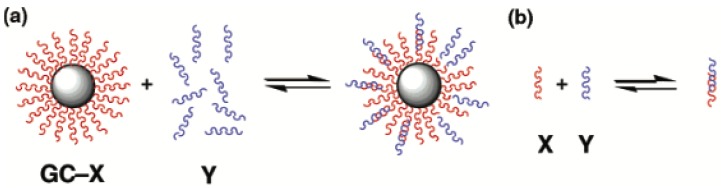
Pictorial representation of hybridization (**a**) on a DNA-modified gold nanoparticle and (**b**) in solution. Hybridization of a complement bearing a dangling end increases the hydrodynamic radius of the nanoparticle and can be followed by dynamic light scattering. X and Y represent two different families of oligonucleotides. Reprinted with the permission from [102]. Copyright 2015 American Chemical Society.

Furst et al. underlined how this process shows a great versatility, and the hybridized DNA structures are able to open many challenging possibilities in the drug delivery field [103]. Figure 5 presents a schema of the hybridization on a DNA-modified gold nanoparticle.

In the following table (Table 2) we have summed up the previously explained strategies, highlighting their advantages and disadvantages.

## 5. Conclusions

The possibilities discussed in this review are not the only available strategies through which we can coat pre-existing particles in order to improve the targeted drug delivery. In the literature, many options in the field of therapeutic delivery to different tissues have been investigated together with a great variety of ligands. Each has its peculiarities and specific sites in which it can act; examples include small molecules such as folic acid or galactose, peptides such as RGD or ATWLPPR, other typologies of proteins or antibodies, and so on. It is clear how in the face of this wide variety of options it is not possible to identify the best coating strategy or ligand molecule that guarantees the best target selectivity, and that the choice is strictly case-dependent. It is indisputable that great strides have been made in the nanomedicine field and their potentialities are clearly evident thanks to in vitro and in vivo tests. Now, the challenge is to be able to realize a scaled-up production of these devices, and especially to be able to focus on the clinical translation of these technologies.

## Figures and Tables

**Table 1:** Main applications of nanoparticles (NPs) drug delivery systems. CNS: central nervous system.

Applications of NPs Drug Delivery Systems	Advantages
Tumors	Passive targeting promoted by enhanced permeability and retention (EPR) effect: accumulation on target site is favored
Diabetes	Less-invasive route for the delivery of insulin and improvement in the drug delivery
Cardiovascular Diseases	Development of regenerative medicine and of the possibility to manage this kind of disease
CNS Injuries	Possibility to overcome biological barriers, reaching the CNS and having a less-invasive delivery system

**Table 2:** Advantages and disadvantages of the available coating strategies for nanoparticles.

Coating Strategies	Advantages	Disadvantages
Polymers	Tunable chain structure Avoids surface agglomeration	Difficulty in polymer functionalization
Cell Membranes	High biocompatibility High specificity thanks to the membrane used	Difficulty in the adhesion of the membrane with the NPs
Proteins and Antibodies	Overcoming opsonization problems due to its physiological peculiarity High selectivity of the final product	Low flexibility in application of the final product
Hybridized DNA Structure	Precise targeting Great versatility	Complex synthesis method
